# Telehealth and Access to HIV Care for Youth: A National Mixed-Methods Study of U.S. HIV Healthcare Provider Practices and Perspectives

**DOI:** 10.1177/23259582261467830

**Published:** 2026-07-17

**Authors:** E. A. Barr, R.P. Momin, Q. Qian, Robin Beach, Mary Catherine Lingwall, M. E. Paul, Hannah Armitage, H. Wu, Thomas P. Giordano

**Affiliations:** 1 Department of Research, Cizik School of Nursing, 16171 University of Texas Health Sciences Center at Houston (UTHealth), Houston, TX, USA; 2 Department of Research, University at Buffalo School of Nursing, Buffalo, NY, USA; 3 Department of Biostatistics & Data Science School of Public Health, 49219 University of Texas Health Science Center at Houston (UTHealth), Houston, TX, USA; 4 Department of Internal Medicine McGovern Medical School, University of Texas Health Science Center at Houston (UTHealth), Houston, TX, USA; 5 Department of Pediatrics, 506057 Baylor College of Medicine, Houston, TX, USA; 6 Department of Medicine, 171841 Baylor College of Medicine, Houston, TX, USA

**Keywords:** HIV, telehealth, adolescent health, patient-provider trust

## Abstract

**Background:**

Youth living with HIV (YWH) face persistent barriers to care engagement and viral suppression. Telehealth expanded post-COVID-19 emergence, offering new possibilities for access, but provider perspectives on its use with YWH are underexplored.

**Methods:**

A national mixed-methods survey captured experiences of 156 HIV care providers. Quantitative data were analyzed using descriptive and inferential statistics; qualitative data were analyzed using reflexive thematic analysis.

**Results:**

Most providers (88.5%) believed telehealth would continue to play a role in HIV care. Reported benefits included improved access (80.7%) and workflow efficiency (64.7%). However, providers were significantly less likely to discuss sensitive topics “often” via telehealth compared to in-person care, including mental health (81.4% vs. 92.9%, p = 0.0008) and sexual health (77.9% vs. 93.6%, p < 0.0001).

**Conclusions:**

While telehealth improves access for YWH, hybrid care models and targeted provider training are needed to address privacy, communication, and trust when discussing sensitive health concerns.

## Plain Language Summary

Telehealth is a valuable tool for improving healthcare access for young people living with HIV (YWH). During the COVID-19 pandemic, clinics started using it more often, creating new ways for patients to connect with their healthcare providers (such as doctors or nurses). In this study, we surveyed 156 HIV care providers across the country to understand their experiences with telehealth. Most providers believe telehealth will remain important for HIV care, especially because it makes it easier for patients to access services and helps clinics work more efficiently. However, we also found that providers were much less likely to have in-depth conversations about sensitive topics like mental health and sexual health with patients during telehealth visits compared to in-person appointments. While telehealth clearly has benefits, our findings show that clinics need to offer a mix of in-person and telehealth options and provide specific training for providers on how to talk about difficult topics virtually. This will help ensure young people get the full support they need to stay healthy.

## Introduction

The HIV epidemic continues to be a significant and persistent global public health crisis that disproportionately impacts adolescents and young adults.^1^ Worldwide, approximately 5 million youth aged 15-24 have HIV,^2^ including both newly diagnosed and previously diagnosed individuals. Youth living with HIV (YWH) face heightened risks of poor health outcomes and continued transmission.^3^ Despite advances in HIV prevention and treatment, various risk factors, including limited access to healthcare, stigma, and lack of awareness, underscore the critical need for targeted interventions and innovative care strategies adapted to the special needs of youth with HIV (YWH).^4^

The onset of the COVID-19 pandemic caused significant disruptions to healthcare delivery systems globally, prompting a rapid shift to telehealth services, including in the care of HIV.^5^ While this transition presented challenges, it also created a pivotal opportunity to reimagine how HIV care is delivered to youth. Telehealth, including synchronous audio and video consultations and audio-only interactions, quickly emerged as an essential tool for maintaining continuity of care when traditional in-person services were unavailable or restricted.^6^

The transition to telehealth was supported by federal policy changes and clinical guidelines, enabling telehealth’s seamless integration into HIV care.^7^ Telehealth has emerged as a preferred modality among YWH, aligning with their communication styles and lifestyles.^6^ Its convenience, privacy, and accessibility offer promising pathways to improve engagement and retention in care, improving access, supporting adherence, and, in some cases, contributing to better viral suppression outcomes.^6^ In the United States, 99% of surveyed HIV providers offered telehealth services, with 47% of visits delivered through this modality.^8^ Among patients, 57% expressed a preference for telehealth over in-person visits.^8^ As telehealth becomes increasingly embedded in care models, understanding its specific impact on populations like YWH and their providers remains essential.

While telehealth has its benefits, it brings challenges such as limited technology access, a lack of private space, and difficulty obtaining timely lab tests. These barriers were especially significant for YWH.^8^ In metropolitan DC, a study focusing on predominantly Black children and YWH, lower rates of laboratory testing with telehealth visits underscored the need for improved infrastructure to support comprehensive remote care.^8^ The reduction of in-person interactions from the uptake in telehealth has raised concerns about the potential impact on care quality and the ability to perform necessary physical assessments.^9^ A 2022 study by Boshara highlighted the digital divide as a critical barrier to telehealth access, particularly among marginalized YWH, suggesting the need for targeted interventions to address these disparities.^10^

Healthcare providers play a pivotal role in shaping how telehealth is implemented and sustained within HIV care models. Understanding how provider training, attitudes, and perceptions influence telehealth delivery is essential to optimizing its effectiveness for YWH. While the benefits of telehealth for patients, particularly YWH, are well-documented, the perceptions, challenges, and experiences of the providers who deliver this care are equally critical to its long-term success. Enhancing provider training and telehealth infrastructure may further strengthen care delivery, but additional research is needed to fully understand and support these dynamics.^6,11^

While telehealth is increasingly used in HIV care, most existing research emphasizes patient-level outcomes, with limited understanding of the perspectives of providers delivering this care, particularly to YWH.^4,6^ This paper examines HIV healthcare provider perspectives on telehealth use when caring for YWH. By exploring provider attitudes, satisfaction levels, and perceptions of telehealth’s benefits and barriers, we aim to uncover critical insights that can guide the effective integration of telehealth into HIV care models. Understanding the factors that influence provider buy-in and utilization is essential for optimizing care delivery and ensuring sustained patient engagement.

## Methods

This study employs a mixed methods embedded design, in which qualitative data play a supporting role within a primarily quantitative study.^12^ The qualitative component was used to contextualize quantitative results.^13^ The study aimed to describe telehealth experiences and examine provider perspectives on the benefits and challenges of telehealth for YWH. The survey instrument was developed by adapting existing surveys (Table 1) and items derived from literature on telehealth care in YWH.^14-28^ Providers ranked benefits and barriers to telehealth use and reported best practices, strategies for maintaining patient-provider trust, and approaches to enhancing HIV care for YWH. Qualitative data were collected concurrently through open-ended (free-text) survey questions to contextualize and expand upon the quantitative findings.^12^ These qualitative responses were provided by the same participants who completed the quantitative survey; no separate interviews were conducted.

**Table 1.** Telehealth Surveys Used in Survey Design

Title of tool/instrument	Citation	Survey information
Baylor College of Medicine Telehealth Survey	Guajardo et al., 2022	A cross-sectional study evaluating the telehealth experiences of providers (n=157) caring for Latinx people with HIV; includes survey instrument developed from validated items.
Swedish Primary Care Physician’s Survey	Pikkemaat et al., 2021	Qualitative study exploring provider (n=198) intentions to use telehealth before and after the COVID-19 pandemic; includes novel survey items tested for construct validity, internal validity, and reliability.
Massachusetts General Hospital Telehealth Provider Survey under “Technical Issues”	Donelan et al., 2019	Qualitative study of provider (n=74) and patient (n=426) perceptions of telehealth experiences compared to in-person medical care; includes over 25 validated Likert measures.

*Note.* Includes instruments that were adapted for use in the survey for this study.

The study included US HIV care providers, specifically Registered Nurses (RNs), Advanced Practice Providers (APPs; i.e., Advanced Practice Nurses (APRNs) and Physician Assistants (PAs)) and Physicians (MD/DOs), who provide care for YWH ages 16-29 years of age. Inclusion criteria required providers be 18 years of age or older, able to read and write in English, and must have provided care in a clinical setting for YWH for 2 or more years.

The survey was disseminated electronically to healthcare providers with a specialization in caring for YWH. Distribution channels included research networks such as the Adolescent Trials Network, Pediatric HIV/AIDS Cohort Studies Network, the International Maternal Pediatric Adolescent AIDS Clinical Trials Network, the Association of Nurses in AIDS Care, the National AIDS Education and Training Center, and the directors of the Centers for AIDS Research. Because the primary aims of this national survey were descriptive, focusing on telehealth use, provider-reported barriers, and provider perceptions of barriers experienced by youth with HIV, sample size was determined based on the precision of estimated proportions rather than a single hypothesis test. Using a conservative estimated proportion of 50%, a sample of 196 providers would yield a 95% confidence interval with a margin of error of approximately ±7%. The final analytic sample included 156 providers, corresponding to a margin of error of approximately ±8%, which was considered acceptable for the primary descriptive aims. Analyses of subgroup differences were considered exploratory. Data were collected between March 10, 2023, and June 20, 2023. Compensation in the form of a gift card was provided.

This study was approved by The Committee for the Protection of Human Subjects (CPHS) at the University of Texas Health Science Center at Houston IRB (protocol HSC-SN-0659) and the Institutional Review Board for Baylor College of Medicine (protocol H-53567), which granted a waiver of written informed consent. Participants were informed about the purpose of the study, and consent was implied by their voluntary completion of the REDCap^29^ survey. The reporting of this study conforms to the Strengthening the Reporting of Observational Studies in Epidemiology (STROBE)^30^ statement (see Supplementary File 1).

Following data collection, responses were screened for eligibility and data quality. Surveys not meeting inclusion criteria or containing incomplete or invalid responses were excluded. Quality-check items embedded in the survey were used to identify potentially invalid entries, and three team members independently reviewed flagged responses. After exclusions, 156 completed surveys met inclusion criteria and were included in the final analytic dataset.

### Data Analysis

Quantitative data were analyzed using descriptive statistics, mean and standard deviations for continuous variables and frequencies and percentages for categorical variables, to summarize the demographics, provider types, and responses to closed-ended questions. Associations between categorical variables were evaluated using Chi-squared tests. To account for multiple comparisons, Bonferroni correction was applied to adjust the significance threshold and control the family-wise error rate.^31^ All statistical analyses were performed using R software (version 4.4.1) and RStudio (version 2024.09.0+375).^32^

The qualitative component was analyzed using Braun and Clarke’s reflexive thematic analysis.^33,34^ This approach aligns with a constructivist epistemology, recognizing that themes are actively generated rather than objectively “emerging” from data.^35^ The team systematically identified, analyzed, and reported patterns (themes) within the data. To reduce individual bias, three team members trained in qualitative analysis independently reviewed and coded the data to gain an in-depth understanding of the content. Prior to using MAXQDA,^36^ three members of the research team independently reviewed the free-text responses and conducted initial open coding to identify preliminary codes and patterns in the data. MAXQDA was then used to organize, manage, and track the coding and thematic analysis. Codes were translated into themes, referring to the original data to ensure that these themes accurately represent the collected information.

The full team, including multidisciplinary healthcare providers, reviewed final themes, ensuring integration between coded extracts and the entire quantitative dataset. Detailed code sheets documenting the evolution of codes into themes, including definitions, inclusion and exclusion criteria for each theme, and illustrative quotes to ensure transparency and replicability of the analysis, providing a clear audit trail.

## Results

### Sample Characteristics

A total of 156 HIV care providers completed the survey. The majority identified as female (77.6%, n=121) and non-Hispanic (87.2%, n=136), with 46.2% (n=72) aged between 40 and 59 years. Most respondents were prescribing providers (71.2%, n=112), including MDs, DOs, and advanced practice providers (APPs) like APRNs, NPs, and PAs; the remaining 28.8% (n=44) were nurses without prescriptive authority (e.g., RNs and MSNs). Providers primarily practiced in urban settings (83.3%, n=130), followed by suburban (13.5%, n=21) and rural areas (3.2%, n=5). Nearly half (44.9%, n=70) had more than 25 years of experience in HIV care. Importantly, over half of providers (51.9%, n=81) reported that 25% or more of their patients were youth aged 16–29 living with HIV, highlighting a strong youth care focus among the sample (Table 2).

**Table 2.** Demographics of HIV Care Providers and Practice Descriptions

​	Physicians	All nurses	All
	or PAs	(APRN, MSN, RN)	(N=156)
	(N=112)	(N=94)	
**Practice size**			
Solo practice	2 (1.8%)	4 (4.3%)	5 (3.2%)
Small group (2-3 providers)	15 (13.4%)	11 (11.7%)	23 (14.7%)
Medium group (4-10 providers)	46 (41.1%)	47 (50.0%)	68 (43.6%)
Large group (11+ providers)	49 (43.8%)	32 (34.0%)	60 (38.5%)
**Geographic location of HIV practice setting**			
Rural	1 (0.9%)	5 (5.3%)	5 (3.2%)
Suburban	14 (12.5%)	12 (12.8%)	21 (13.5%)
Urban	97 (86.6%)	77 (81.9%)	130 (83.3%)
**Practicing in a state without Medicaid expansion**			
No	76 (67.9%)	66 (70.2%)	107 (68.6%)
Yes	36 (32.1%)	28 (29.8%)	49 (31.4%)
**Years spent as an HIV provider, including training**			
<5	10 (8.9%)	27 (28.7%)	37 (23.7%)
5<10	11 (9.8%)	6 (6.4%)	17 (10.9%)
10-24	46 (41.1%)	26 (27.7%)	57 (36.5%)
>25	45 (40.2%)	46 (48.9%)	70 (44.9%)
**Age**			
<39	36 (32.1%)	34 (36.2%)	54 (34.6%)
40-59	54 (48.2%)	39 (41.5%)	72 (46.2%)
60 or older	21 (18.8%)	20 (21.3%)	29 (18.6%)
Prefer not to answer	1 (0.9%)	1 (1.1%)	1 (0.6%)
**Hispanic or Latino/a**			
No	94 (83.9%)	85 (90.4%)	136 (87.2%)
Yes	16 (14.3%)	7 (7.4%)	18 (11.5%)
Prefer not to answer	2 (1.8%)	2 (2.1%)	2 (1.3%)
**Sex**			
Female	83 (74.1%)	80 (85.1%)	121 (77.6%)
Male	27 (24.1%)	11 (11.7%)	31 (19.9%)
Other/Prefer not to answer	2 (1.8%)	3 (3.3%)	4 (2.5%)
**% of patients with HIV in their practice are youth 16-29 yrs**			
0 - 24%	56 (50.0%)	44 (46.8%)	75 (48.1%)
25 - 75%	38 (33.9%)	38 (40.4%)	57 (36.5%)
>75%	18 (16.1%)	12 (12.8%)	24 (15.4%)
**% of youth patients with HIV with transportation challenges**			
0 - 24%	33 (29.5%)	26 (27.7%)	42 (26.9%)
25 - 75%	58 (51.8%)	49 (52.1%)	86 (55.1%)
>75%	21 (18.8%)	19 (20.2%)	28 (17.9%)
**Providing primary care for this % of youth patients with HIV**			
0 - 24%	8 (7.1%)	5 (5.3%)	10 (6.4%)
25 - 75%	34 (30.4%)	34 (36.2%)	50 (32.1%)
>75%	70 (62.5%)	55 (58.5%)	96 (61.5%)
**% of youth patients with HIV with limited English proficiency?**			
0 - 24%	42 (37.5%)	36 (38.3%)	59 (37.8%)
25 - 75%	19 (17.0%)	13 (13.8%)	23 (14.7%)
>75%	51 (45.5%)	45 (47.9%)	74 (47.4%)
**% of time you use a translator service or trained bilingual staff?**			
0 - 24%	9 (8.0%)	10 (10.6%)	13 (8.3%)
25 - 75%	11 (9.8%)	6 (6.4%)	12 (7.7%)
>75%	92 (82.1%)	78 (83.0%)	131 (84.0%)

*Note.* Demographic descriptions of providers and their practice. PA = Physician Assistant, APRN = Advanced Practice Registered Nurse, RN= Registered Nurse, MSN= Master of Science in Nursing.

Although no differences reached statistical significance, several trends emerged between nurses and physicians/PAs. Nurses were slightly more likely to report over 25 years of experience in HIV care (48.9% vs. 40.2%) and were more frequently based in rural settings (5.3% vs. 0.9%). While the majority of both groups identified as female, this was more pronounced among nurses (85.1%) compared to physicians/PAs (74.1%) (p = 0.078) (Table 2).

### Benefits and Barriers of Telehealth for YWH

Survey responses indicated that most providers believed telehealth improves access to care (80.7%, n=117), that it will play a role in the future of HIV care (88.5%, n=138), and that telehealth improved daily workflow and efficiency in HIV care (Table 3). Additionally, a majority of the sample (80.7%, n=117), supported expanding telehealth services for YWH. Providers identified several other key benefits, including greater scheduling flexibility for patients (36.6%, n=53) and improved convenience for providers (40.0%, n=58). In a separate survey item, providers were asked to rank patient-specific benefits of telehealth. The most frequently selected top-ranked benefit was decreased practical barriers to care, such as transportation or childcare (66.9%, n=97). Providers reported a median of 25% of visits conducted via telehealth (mean 32.1%), with a preference for video-based modalities rather than audio-only or text-based options, as demonstrated by most providers agreeing or strongly agreeing with the statement that video visits should be expanded for YWH (n=117, 80.7%).

**Table 3.** Ranked Telehealth Benefits and Barriers by Providers

Category	Top 3 ranked	Bottom 3 ranked
Patient benefits	1. Decreases barriers to care (i.e. transportation, childcare, etc.) n=97 (66.9%)	4. Protects privacy n=38 (26.2%)
	2. Patients prefer flexibility n=53 (36.6%)	5. Protects the health of patients n=47 (32.4%)
	3. Saved time for patients n=44 (33.3%)	6. Increased ability to build patient-provider trust n=53 (36.6%)
Provider benefits	1. Convenient for providers n=58 (40.0%)	4. Protects patients/staff n=33 (22.8%)
	2. Improves clinic efficiency n=53 (36.6%)	5. Increases patient-provider trust n=63 (43.4%)
	3. Fewer no-show/canceled appointments n=40 (27.6%)	6. Favorable compensation n=64 (44.1%)
Patient barriers	1. Technical difficulties for the patient n=51 (35.2%)	4. Concerns about patient privacy n=31 (21.4%)
	2. Inability for patients to complete paperwork/meet with support staff n=31 (21.4%)	5. Decreased ability to build trust with provider n=39 (26.9%)
	3. Access to technology n=23 (15.9%)	6. Unfavorable insurance coverage n=54 (37.2%)
Provider barriers	1. Challenges obtaining labs, STI tests, or other clinical tests n=55 (37.9%)	4. Obtaining vital signs n=33 (22.8%)
	2. Inability to perform a complete physical exam n=38 (26.2%)	5. Patient may be distracted or not in an appropriate setting n=36 (24.8%)
	3. Barrier to providing vaccines n=40 (27.6%)	6. Challenges in building patient-provider trust n=59 (40.7%)

*Note.* Describes the benefits of in-person vs. telehealth, as reported by providers (n=145).

While 45.5% of providers (n=71) were neutral about the impact of telehealth on patient engagement, 39.7% (n=62) believed it enhanced engagement and adherence, and 14.7% (n=23) believed it had a negative impact. Providers were more likely to view telehealth as appropriate for youth already engaged in care (37.8%, n=59) compared to those less engaged (7.1%, n=11). Nonetheless, most reported offering telehealth regardless of a patient’s engagement level (Table 4).

**Table 4.** Telehealth Use and Preferences by Provider Type

​	Physicians or PAs (N=112)	All nurses (APRN, MSN, RN) (N=94)	All (N=156)
**% of youth patients with HIV who do not have access to the EQUIPMENT necessary to conduct video visits? (e.g. smart phone, computer, or tablet etc.)**			
0 - 24%	82 (73.2%)	72 (76.6%)	118 (75.6%)
25 - 75%	24 (21.4%)	18 (19.1%)	32 (20.5%)
>75%	6 (5.4%)	4 (4.3%)	6 (3.8%)
**% of visits youth patients with HIV used VIDEO telehealth BEFORE the COVID-19 pandemic caused shelter in place orders in the U.S. (March 15, 2020)**			
< 5%	101 (90.2%)	80 (85.1%)	136 (87.2%)
5 - 25%	7 (6.3%)	9 (9.6%)	12 (7.7%)
26 - 50%	2 (1.8%)	5 (5.3%)	6 (3.8%)
51 - 75%	1 (0.9%)	0 (0%)	1 (0.6%)
>75%	1 (0.9%)	0 (0%)	1 (0.6%)
Mean (SD)	31.1 (22.6)	34.8 (22.8)	32.1 (22.0)
Median [Min, Max]	25.0 [0, 100]	26.5 [0, 100]	25.0 [0, 100]
**In your IDEAL practice, what % of visits do you conduct virtually using telehealth? (Categorized)**			
0%	5 (4.5%)	4 (4.3%)	6 (3.8%)
1 - 25%	61 (54.5%)	41 (43.6%)	80 (51.3%)
26 - 40%	17 (15.2%)	14 (14.9%)	22 (14.1%)
41 - 74%	21 (18.8%)	28 (29.8%)	38 (24.4%)
75 - 100%	8 (7.1%)	7 (7.4%)	10 (6.4%)
**Who is offered telehealth in your clinical practice?**			
No one, we do not offer Telehealth visits	5 (4.5%)	4 (4.3%)	7 (4.5%)
Everyone equally	57 (50.9%)	49 (52.1%)	79 (50.6%)
Patients who are LESS engaged-in-care	8 (7.1%)	7 (7.4%)	11 (7.1%)
Patients who are MORE engaged-in-care	42 (37.5%)	34 (36.2%)	59 (37.8%)
**Telehealth will play a role in the future of HIV care.**			
Disagree	4 (3.6%)	2 (2.1%)	4 (2.6%)
Neutral	7 (6.3%)	12 (12.8%)	14 (9.0%)
Agree	101 (90.2%)	80 (85.1%)	138 (88.5%)
**Patients who use video telehealth are more engaged in care through both medication adherence and being seen by a provider more often.**			
Disagree	18 (16.1%)	14 (14.9%)	23 (14.7%)
Neutral	51 (45.5%)	34 (36.2%)	71 (45.5%)
Agree	43 (38.4%)	46 (48.9%)	62 (39.7%)
**Telehealth has the potential to improve my daily workflow/efficiency.**			
Disagree	10 (8.9%)	9 (9.6%)	15 (9.6%)
Neutral	29 (25.9%)	23 (24.5%)	40 (25.6%)
Agree	73 (65.2%)	62 (66.0%)	101 (64.7%)

*Note.* Telehealth use and experiences of HIV providers caring for youth with HIV.

#### Technology

Despite its advantages, providers identified significant barriers to telehealth implementation for both patients and providers (Table 3). The most frequently cited challenge was patient-side technical difficulties, such as unreliable internet, lack of access to a smartphone or laptop, or limited digital literacy, with 35.2% (n=51) of providers ranking this as the top patient barrier. Additionally, 26.9% (n=30) reported that more than 25% of their youth patients lacked the necessary equipment for video visits. Only 26.2% (n=38) of providers felt that video visits allowed patients to feel more comfortable being open, and just 17.9% (n=26) agreed that video visits improved the overall quality of care (Table 5).

**Table 5.** Perceptions of Video Telehealth: Benefits and Barriers

​	Everyone (N=145)
**Video visits increase patient privacy**	
Strongly disagree	10 (6.9%)
Disagree	28 (19.3%)
Neutral	69 (47.6%)
Agree	33 (22.8%)
Strongly agree	5 (3.4%)
**Video visits improve quality of patient care**	
Strongly disagree	8 (5.5%)
Disagree	46 (31.7%)
Neutral	65 (44.8%)
Agree	21 (14.5%)
Strongly agree	5 (3.4%)
**Video visits reduce patients’ anxiety about being seen at an HIV clinic**	
Strongly disagree	5 (3.4%)
Disagree	9 (6.2%)
Neutral	30 (20.7%)
Agree	81 (55.9%)
Strongly agree	20 (13.8%)
**Video visits increase the number of times I can interact with my patients**	
Strongly disagree	5 (3.4%)
Disagree	25 (17.2%)
Neutral	20 (13.8%)
Agree	77 (53.1%)
Strongly agree	18 (12.4%)
**Video visits improve access to care**	
Strongly disagree	7 (4.8%)
Disagree	4 (2.8%)
Neutral	17 (11.7%)
Agree	80 (55.2%)
Strongly agree	37 (25.5%)
**Video visits reduce the amount of time patients spend getting to/from visits**	
Strongly disagree	4 (2.8%)
Disagree	3 (2.1%)
Neutral	5 (3.4%)
Agree	64 (44.1%)
Strongly agree	69 (47.6%)
**Video visits allow patients to feel more comfortable being open with their provider**	
Strongly disagree	7 (4.8%)
Disagree	26 (17.9%)
Neutral	74 (51.0%)
Agree	29 (20.0%)
Strongly agree	9 (6.2%)
**Video visits take less time for the patients than in person visits**	
Strongly disagree	4 (2.8%)
Disagree	5 (3.4%)
Neutral	21 (14.5%)
Agree	80 (55.2%)
Strongly agree	35 (24.1%)

*Note.* N=145 of the total N completed the ***Telehealth Benefits and Barriers*** survey.

#### Access to Care & Engagement in Care

Although most providers believed that telehealth improved access and convenience, 66.2% (n=103) felt that communication was more effective in person. Providers acknowledged that telehealth allowed for more frequent check-ins and timely adherence support, but only 39.7% (n=62) agreed that it meaningfully enhanced engagement, while 45.5% (n=71) were neutral and 14.7% (n=23) disagreed.

Table 4 compares responses between providers with a primarily medical model background (physicians and PAs) and those with a nursing background (nurses and APRNs). Perceptions of telehealth’s impact on engagement and care outcomes were mixed across both groups. Overall, 45.5% (n = 71) of providers were neutral about its influence on youth engagement, while 39.7% (n = 62) believed youth who used telehealth were more engaged and adherent, and 14.7% (n = 23) believed they were less engaged. Nurses were slightly more likely than physicians/PAs to view telehealth as enhancing engagement (48.9%, n = 46 vs. 38.4%, n = 43), while physicians/PAs were more likely to express neutrality (45.5%, n = 51 vs. 36.2%, n = 34). Although these differences were not statistically significant, they suggest subtle distinctions in perception by provider type. Regardless of background, the overwhelming majority agreed that telehealth would continue to play a role in the future of HIV care delivery, indicating widespread support for its continued integration.

#### Communication and Care Content

Providers reported that core components of care, such as medication adherence, lab results, personal life stressors, substance use, mental health, and sexual health, were addressed in both telehealth and in-person encounters (Table 3). However, there were clear differences in how frequently sensitive topics were discussed. Providers were significantly less likely to report discussing psychosocial and sensitive topics “often” during telehealth visits. For example, home and work life were discussed often in 78.8% of telehealth visits versus 94.9% of in-person visits (p < 0.0001). Similar differences were noted for substance use (72.1% vs. 92.3%, p < 0.0001), mental health (81.4% vs. 92.9%, p = 0.0008), and sexual health (77.9% vs. 93.6%, p < 0.0001). These differences remained statistically significant after applying a Bonferroni correction (adjusted p < 0.0083), underscoring a consistent pattern of reduced depth in virtual visits.

#### Clinical Limitations and Care Quality

Providers reported that telehealth was less effective for complex clinical encounters. Although telehealth was considered useful for routine follow-up care, including medication management and adherence counseling, many providers highlighted the limitations in conducting physical examinations or collecting laboratory specimens remotely. Even when providers covered essential topics such as medication adherence and test results, they were significantly less likely to report discussing psychosocial or sensitive topics during telehealth visits compared to in-person visits (Table 6). Providers were less likely to discuss home and work life (78.8% telehealth vs. 94.9% in-person, p < 0.0001), substance use (72.1% vs. 92.3%, p < 0.0001), mental health (81.4% vs. 92.9%, p = 0.0008), and sexual health (77.9% vs. 93.6%, p < 0.0001) during remote encounters (Table 6). These differences remained statistically significant after applying a Bonferroni correction.^31^

**Table 6.** Frequency of Health Topics Discussed During In-Person vs. Telehealth Visits

*Question: How often do you talk about the following in an in-person visit and in a telehealth visit?*				
Question:	Answers^1^	Visit type		P-value
		In-person visit (N=156)	Telehealth^2^ (N=312)	
Adherence to medications	<=Sometimes	3 (1.9%)	19 (6.1%)	0.0614
	>=Often	153 (98.1%)	293 (93.9%)	
Test results (e.g. labs, imaging)	<=Sometimes	4 (2.6%)	22 (7.1%)	0.05355
	>=Often	152 (97.4%)	290 (92.9%)	
Home and work life (e.g. housing status, life stressors)	<=Sometimes	8 (5.1%)	66 (20.2%)	<0.0001*
	>=Often	148 (94.9%)	246 (78.8%)	
Substance use (e.g. tobacco, alcohol, drug use)	<=Sometimes	12 (7.7%)	87 (27.9%)	<0.0001*
	>=Often	144 (92.3%)	225 (72.1%)	
Mental health	<=Sometimes	11 (7.1%)	58 (18.6%)	0.0008*
	>=Often	145 (92.9%)	254 (81.4%)	
Sexual health (e.g. STIs, PrEP, birth control, condom use)	<=Sometimes	10 (6.4%)	69 (21.1%)	<0.0001*
	>=Often	146 (93.6%)	243 (77.9%)	

*Significant after the Bonferroni multiple testing adjustment (p=0.05/6 = 0.0083).

^1^Notes. Original response options were: “Never,” “Rarely,” “Sometimes,” “Often,” and “Always.” Responses were collapsed into two categories for analysis: *<=Sometimes* includes “Never,” “Rarely,” or “Sometimes,” and *>=Often* includes “Often” or “Always.”

^2^Telehealth includes audio and video telehealth combined.

Providers shared mixed concerns about the effects of telehealth on relational aspects of health care, such as quality of interaction, the experience of social stigma, or trust within the patient-provider relationship. While 69.7% (n=101) of providers agreed that video visits helped reduce patient anxiety about being seen at an HIV clinic, only 65.5% (n=95) felt telehealth improved opportunities for patient interaction (Table 5).

#### Policy & Reimbursement

Another critical barrier involved uncertainty regarding long-term reimbursement for telehealth. While federal and state-level regulations during the COVID-19 pandemic expanded reimbursement, many providers expressed concern about the sustainability of these policies. Over one-third of respondents (37.2%, n = 54) ranked unfavorable insurance coverage or inadequate compensation among the top three provider-side barriers (Table 3). These concerns were especially pronounced among providers working in smaller, resource-limited clinics.

### Integrated Qualitative Results

Of the 156 providers included in the analytic sample, 137 provided at least one response to the open-ended survey questions, while 19 did not provide free-text responses**.** Qualitative findings both corroborated and expanded upon quantitative results, offering additional context, nuance, and in some cases highlighting tensions not fully captured by survey responses. Reflexive thematic analysis revealed three central themes: *Structural and Technical Barriers to Telehealth Implementation, Enhancing Access and Engagement in Care,* and *Challenges and Opportunities in Building Trust and Connection*. These themes help contextualize and deepen interpretation of the survey data, supporting best practices for integration in mixed-methods research. Findings from the synthesis of the quantitative and qualitative data are presented here.

### Structural and Technical Barriers to Telehealth Implementation

Consistent with survey results showing technical challenges as a leading barrier (Table 3), providers described unstable internet, limited access to devices, and low digital literacy among patients as frequent obstacles to effective care. As one family nurse practitioner with 5–25 years of experience explained, “Telehealth helps keep patients with transportation, location, and work barriers engaged in care.” However, others noted that infrastructure gaps undermined access, particularly in rural areas. “I see these limitations primarily on the patient-side, with poor cellular connectivity in rural portions of our state,” shared a physician with 5–25 years of experience. These qualitative perspectives reinforce the survey results that one in four providers reported more than 25% of their youth patients lacked the necessary equipment for video visits, and help explain the mixed quantitative perceptions of engagement. Providers in this survey expressed that while telehealth may reduce logistical barriers and support autonomy for some youth, its effectiveness depends on individual readiness, self-management capacity, and environmental context.

### Enhancing Access and Engagement in Care

Many providers emphasized that telehealth could support engagement by reducing barriers and increasing patient autonomy, echoing quantitative data where 39.7% of respondents believed telehealth users were more engaged and adherent (Table 4). One nurse practitioner with under 5 years of experience shared, “Electronic health care allows patients to feel more empowered in their health outcomes.” Similarly, a physician (<5 years of experience) reported that telehealth “has been excellent in getting [patients] back to clinic and engaging in their own health.” Several providers noted that electronic tools enhanced patient engagement, particularly through real-time access to appointments and lab results. As one nurse practitioner with under 5 years of experience shared, “I think patients really appreciate the ability to see their appointments and results in real time.” Another provider noted that the *“*patient portal is useful to some patients as a way to communicate concerns/questions that they might forget during their visit” (physician, 5–25 years of experience). These tools were viewed as supporting autonomy and helping youth stay engaged between visits.

However, this optimism was tempered by concerns that some youth may disengage without strong self-management skills. “Although we do everything we can to make it easy for patients to go in for labwork…it takes a lot of reminders and sometimes doesn’t happen,” shared a physician with more than 25 years of experience. Another provider noted the distractions in non-private settings: “I have asked the patient if they were comfortable talking where they were… only to find out they were in the supermarket or laundromat and definitely not paying attention. I worried about the effectiveness of the visit and about HIPAA” (advanced practice nurse, over 25 years of experience). These reflections help explain the quantitative finding that providers were significantly less likely to discuss sensitive topics such as mental health or sexual health via telehealth (Table 6), suggesting that environmental distractions, lack of privacy, and competing demands may limit the depth of these conversations.

### Challenges and Opportunities in Building Trust and Connection

Trust, comfort, and emotional connection, key to sensitive HIV care, emerged as central concerns in telehealth delivery. While quantitative data showed only 26.2% of providers believed video visits helped patients be more open (Table 5), qualitative responses offered a more nuanced view. Several providers described feeling limited in their ability to build intimacy virtually, particularly during emotionally or clinically complex conversations. As one provider described, “It is a little more difficult to talk about more sensitive topics virtually, especially if there are connection issues” (physician, 5–25 years of experience).

Yet others found that the privacy of home allowed for more honest disclosure. A physician with over 25 years of experience remarked, “I’ve had several patients disclose information during a call that they had not previously disclosed in person.” This contrast mirrors the quantitative ambivalence on communication quality, where 66.2% felt in-person visits were better, but many acknowledged telehealth’s convenience and its role in reducing stigma (Table 5).

Providers also emphasized the need for enhanced training and support. A physician assistant with under 5 years of experience called for “additional training on best methods for conducting virtual visits, especially when assessing mental health concerns.” Another physician (>25 Years of experience) suggested patient-side training: “how to find and turn on the microphone and camera, etc.” Taken together, these qualitative perspectives deepen interpretation of the quantitative findings by revealing that telehealth may both facilitate and constrain trust and disclosure, depending on privacy, connection quality, and the relational dynamics of the encounter.

## Discussion

This study provides a nuanced view of how telehealth is experienced by providers caring for youth with HIV, highlighting both its potential to improve access and its limitations in supporting relational and complex aspects of care. Results from this study suggest that providers view telehealth as a modality that improves access to care by reducing transportation and scheduling barriers, consistent with literature describing telehealth as an important tool for maintaining continuity of HIV care during the COVID-19 pandemic.^8,11^ However, the findings also highlight persistent digital barriers. Over one-third of providers cited patient-side technical issues as a major barrier, and more than one-fourth reported that a quarter or more of their YWH patients lacked adequate equipment for video visits. While prior literature acknowledged logistical challenges during rapid telehealth adoption,^8^ our data quantify the extent to which digital readiness remains uneven. These results suggest that telehealth’s access benefits are conditional and dependent on structural supports. This suggests that telehealth effectiveness is shaped not only by availability, but by digital literacy, stability of internet access, and competing demands within patients’ environments. Similar results have been reported in large observational studies of primary care visits, which demonstrate that telehealth outcomes vary by visit modality and patient socioeconomic status (SES), with video visits less accessible among lower-SES populations.^37^ Patterns also vary by provider background; nurses in this sample were more likely than physicians to work in rural settings and to report over 25 years of experience, which may influence how telehealth is implemented and how patient engagement is supported. Together, these patterns underscore the need for digital equity initiatives, including device access and broadband infrastructure, to support inclusive care delivery. Similar disparities in telehealth perceptions have been reported among patients with chronic conditions, particularly among older or lower-socioeconomic populations, underscoring how technology access and digital literacy shape engagement with virtual care.^38^

Providers also reported that telehealth mitigated logistical barriers for YWH, and digital tools such as electronic health records (EHRs) and patient portals were identified as valuable for promoting engagement. These platforms allow youth to access health information, manage appointments, and communicate with providers, potentially fostering greater autonomy in managing their health. At the same time, providers expressed concern regarding telehealth’s impact on engagement and long-term retention. The results align with adolescent HIV intervention literature emphasizing that retention is influenced by multi-level structural and relational factors beyond access alone.^1,4^ For example, the SMILE initiative demonstrated improvements in timely linkage to care among newly diagnosed youth, yet sustained engagement required continued support mechanisms.^3,4^ Research examining the transition from pediatric to adult HIV care similarly highlights the importance of coordinated systems and trust-building in maintaining engagement.^3^ These results align with prior literature by demonstrating that while telehealth reduces logistical barriers, it does not independently resolve retention challenges. This variability likely reflects differences in patient readiness, self-management capacity, and the level of external support available to youth. Providers noted that youth with weaker self-management skills were more likely to struggle with engagement in virtual contexts, reinforcing developmental considerations described in prior adolescent HIV care research.^1,3^ Overall, providers viewed telehealth as a promising strategy to enhance access, autonomy, and engagement in care, while recognizing that meaningful engagement requires more than convenience alone.

A central finding of this study was that providers were significantly less likely to discuss sensitive topics, including mental health, sexual health, and substance use, during telehealth visits compared to in-person encounters. The finding contrasts with broader telehealth satisfaction literature reporting high patient satisfaction with virtual care modalities.^5^ While telehealth may be acceptable and convenient, satisfaction does not necessarily reflect the depth of clinical dialogue. Our results align more closely with guidance on telehealth consultations with adolescents, which emphasizes confidentiality, privacy assessment, and intentional rapport-building in virtual settings.^9^ Adolescents may lack private spaces during telehealth visits, limiting open disclosure.^9^ This may reflect the intersection of developmental stage, privacy constraints, and provider uncertainty in navigating sensitive conversations without visual or environmental cues. The reduced frequency of sensitive-topic discussions observed in this study suggests that telehealth may constrain psychosocial assessment unless providers are supported with structured communication strategies and privacy protocols. At the same time, some providers described enhanced openness when youth connected from familiar environments, highlighting the context-dependent nature of telehealth communication.^9^ These integrated results suggest that telehealth may either facilitate or limit disclosure depending on environmental and relational factors.

Providers also emphasized that telehealth alone is insufficient for delivering high-quality HIV care. Many noted its limitations for physical assessments, laboratory testing, and addressing psychosocial needs. Across both quantitative and qualitative findings, providers favored a hybrid care model, combining telehealth with periodic in-person visits. This perspective aligns with reports describing telehealth implementation in HIV care as complementary rather than substitutive during the COVID-19 pandemic.^11^ Providers who incorporated telehealth within structured hybrid care models reported more positive experiences, suggesting that intentional integration may improve care delivery for YWH.

Finally, providers identified a need for structural and training support to optimize telehealth delivery. Recommendations included developing best practices for virtual trust-building, ensuring patient privacy, and standardizing workflows for laboratory coordination and mental health evaluation. Investing in provider and patient training may strengthen the quality of virtual care and reduce disparities in telehealth engagement. These integrated findings suggest that telehealth effectiveness is not solely determined by modality, but by the interaction of structural access, developmental context, and trust-building provider communication strategies.

### Limitations

This study has several limitations. First, the sample was limited to providers in the United States who responded to an online survey, which may introduce selection bias; those more comfortable with technology or with stronger opinions about telehealth may have been more likely to participate. While diverse in discipline, geography, and years of experience, the sample may not represent all provider perspectives, particularly from rural or under-resourced settings where telehealth infrastructure is more limited. This limited our ability to examine differences in telehealth barriers by rural versus urban practice characteristics, for example. Second, the data were self-reported and may be subject to recall or social desirability bias, especially regarding sensitive topics such as provider comfort or patient disclosure during virtual visits. Additionally, although the mixed-methods design allowed for nuanced exploration of telehealth experiences, the qualitative data were drawn from open-ended survey responses rather than in-depth interviews or focus groups, which may limit the depth of insight. Finally, the study was conducted in the post-acute phase of the COVID-19 pandemic, during which telehealth policies and workflows were in flux. Provider experiences and attitudes may shift over time as regulatory, reimbursement, and technological landscapes continue to evolve. Future longitudinal and implementation-focused studies are needed to assess how telehealth practices and impacts change in more stable healthcare environments.

## Conclusion

This study highlights both the promise and the complexity of telehealth in HIV care for youth. Providers widely endorsed telehealth as a tool to enhance access, reduce logistical barriers, and support continuity of care, especially for youth who face challenges with transportation, school, or work. However, this optimism was tempered by persistent barriers, including unequal access to technology, limitations in addressing sensitive topics, and concerns about diminished trust and intimacy in virtual settings. Addressing these barriers will be critical to ensure that telehealth can meet the comprehensive needs of YWH, particularly in managing the multifaceted nature of their care. To realize the full potential of telehealth for youth with HIV, targeted training in virtual communication, investment in digital infrastructure, and clear clinical protocols for hybrid care are essential. As healthcare systems evolve, centering the voices and needs of providers and young people will be key to designing effective, responsive, and youth-affirming care models.

## Supplemental Material

Supplemental material - Telehealth and Access to HIV Care for Youth: A National Mixed-Methods Study of U.S. HIV Healthcare Provider Practices and Perspectives Supplemental material for Telehealth and Access to HIV Care for Youth: A National Mixed-Methods Study of U.S. HIV Healthcare Provider Practices and Perspectives by E. A. Barr, R.P. Momin, Q. Qian, R. Beach, M. Lingwall, M. E. Paul, H. Armitage, H. Wu, T. P. Giordano in Journal of the International Association of Providers of AIDS Care (JIAPAC)

Supplemental material - Telehealth and Access to HIV Care for Youth: A National Mixed-Methods Study of U.S. HIV Healthcare Provider Practices and Perspectives Supplemental material for Telehealth and Access to HIV Care for Youth: A National Mixed-Methods Study of U.S. HIV Healthcare Provider Practices and Perspectives by E. A. Barr, R.P. Momin, Q. Qian, R. Beach, M. Lingwall, M. E. Paul, H. Armitage, H. Wu, T. P. Giordano in Journal of the International Association of Providers of AIDS Care (JIAPAC)
