# Corrigendum to “Telehealth and Access to HIV Care for Youth: A National Mixed-Methods Study of U.S. HIV Healthcare Provider Practices and Perspectives”

**DOI:** 10.1177/23259582261486118

**Published:** 2026-08-30

Barr EA, Momin RP, Qian Q, et al. Telehealth and Access to HIV Care for Youth: A National Mixed-Methods Study of U.S. HIV Healthcare Provider Practices and Perspectives. *Journal of the International Association of Providers of AIDS Care*. 2026; 25: 1–13. 10.1177/23259582261467830

In this article, the author names have been updated to display the full author names as follows: Emily Anne Barr, Rashmi P. Momin, Qian Qian, Robin Beach, Mary Catherine Lingwall, Mary E. Paul, Hannah Armitage, Hulin Wu, and Thomas P. Giordano. The associated metadata has also been updated to ensure accurate indexing.

The online version of the article has been updated accordingly.

