# Supplementary material for: Telehealth and Access to HIV Care for Youth: A National Mixed-Methods Study of U.S. HIV Healthcare Provider Practices and Perspectives
Source: J Int Assoc Provid AIDS Care. 2026 Jul 17;25:23259582261467830. doi: 10.1177/23259582261467830 (PMC13379646; doi:10.1177/23259582261467830)
Supplement: Supplemental material - Telehealth and Access to HIV Care for Youth: A National Mixed-Methods Study of U.S. HIV Healthcare Provider Practices and Perspectives [file sj-pdf-2-jia-10.1177_23259582261467830.pdf]

Experience with Telehealth among HIV Providers in the Era of COVID-19

Dear Healthcare Provider,

We are conducting a survey of nursing and medical care providers who take care of youth living with HIV (YLWH) age 16-29 in the United States.

The survey assesses provider attitudes and beliefs related to Telehealth.

Your participation is voluntary, and you can stop participating at any time. All your responses are private and confidential; we will not share individual data with your supervisor, program director, or employer.

By taking the survey, you agree to take part in the study. You may only take the survey once. The survey will take 20-25 minutes to complete. You will have the option to save and complete at a later time if you are unable to complete the survey once you begin.

For your participation in the study you will receive a $25 gift card. You will need to enter an institutional work email that we can verify for compensation at the end of the survey. Your email will not be connected to your survey and wll only be used to send you the gift card.

PLEASE DO NOT TAKE THIS SURVEY IF YOU ARE NOT A CLINICAL PROVIDER

You will not be compensated for your time if you are not eligible.
If you have questions about your rights as a research subject, contact the Institutional Review Board at 713-500-7943.

If you have questions regarding the project, please email Dr. Barr at emily.barr@uth.tmc.edu or Dr. Paul at mpaul@bcm.edu.

Click "Submit" to begin the survey

Eligibility

The following questions will ensure you meet the inclusion criteria for our study. If you do not, the survey will end. If you are not able to provide a work email - we will not be able to compensate you with the gift card.

A GMAIL or other non-work email will need to be verified with your place of emplyement before compensation. So, if you are not eligible please do not complete the survey.

Thank you again for your time

What is your current role? Check all that apply.	☐ MD<br>☐ DO<br>☐ PA<br>☐ RN<br>☐ NP<br>☐ PNP<br>☐ FNP<br>☐ ANP<br>☐ WHNP<br>☐ CNM<br>☐ Masters prepared nurse but not a practitioner (MSN, MS)<br>☐ Other clinical provider<br>☐ I am not a clinical provider
Have you been providing HIV care for about 20 or more youth living with HIV between the ages of 16 and >/= 29 years for at least 2 years?<br>(This could be in a clinical setting where the care is shared or you cover this younger adult age group with a team of clinical providers)	○ Yes<br>○ No
Was this participant eligible for the study? (ie did they complete this section and start the demographics section?)	○ Yes<br>○ No

Provider and Patient Demographics

SECTION 1/5
This first section will help us understand more about your clinical practice and experience.

Which of these best describes your primary employment setting? Check all that apply.	☐ Academic Health Center or Research Program in a University Setting (med school, nursing school, etc.)<br>☐ Correctional Facility<br>☐ Federally Qualified Health Center (FQHC)<br>☐ Family Planning Clinic<br>☐ HMO/Managed Care Organization<br>☐ Hospital-based Clinic<br>☐ Indian Health Services/Tribal Clinic<br>☐ Maternal/child Health Clinic<br>☐ Mental Health Clinic<br>☐ Ryan White-funded Clinic<br>☐ STD/STI Clinic<br>☐ Military or Veteran's Health Facility<br>☐ Private Practice Clinic<br>☐ State or Local Health Department<br>☐ Other
If "other", please specify:	______________________
What is your specialty? Check all that apply.	☐ Infectious Diseases<br>☐ HIV<br>☐ Internal Medicine<br>☐ Family Medicine<br>☐ Pediatrics<br>☐ Obstetrics/Gynecology<br>☐ Immunology<br>☐ Adolescent Health<br>☐ Other
If "other", please specify:	______________________
What is your practice size?	○ Solo practice<br>○ Small group (2-3 providers)<br>○ Medium group (4-10 providers)<br>○ Large group (11+ providers)
What is the geographic location of your primary practice?	○ Urban<br>○ Suburban<br>○ Rural
What is the zip code of your primary practice?<br><br>(Must be a US-based practice)	______________________
Including the years you spent in training, how many years have you been working as an outpatient HIV provider?	○ >25<br>○ 16-24<br>○ 10-15<br>○ 5-9<br>○ < 5

What is your age?	◯ 80 or older<br>◯ 70-79<br>◯ 60-69<br>◯ 50-59<br>◯ 40-49<br>◯ 30-39<br>◯ 29 or younger<br>◯ Prefer not to answer
What is your race? Check all that apply.	☐ American Indian or Alaska Native<br>☐ Asian<br>☐ Black or African-American<br>☐ Black or African<br>☐ Native Hawaiian or other Pacific Islander<br>☐ White<br>☐ Other<br>☐ Prefer not to answer
If "other", please specify:	____________________
Are you of Hispanic or Latino/a origin?	◯ Yes<br>◯ No<br>◯ Prefer not to answer
What is your gender?	◯ Female<br>◯ Male<br>◯ Trans Female<br>◯ Trans Male<br>◯ Non-Binary<br>◯ Other<br>◯ Prefer not to answer
If "other", please specify:	____________________

These questions will help us understand more about the youth patients living with HIV (YLWH) age 16-29 that you serve.

We will use YLWH to refer to this subset of your patients for the rest of the survey.

If you are unsure, please provide your best estimate.

Among all of the patients with HIV that you care for, what percentage are youth ages 16-29 living with HIV (YLWH)?

- ◯ >75%
- ◯ 51 - 75%
- ◯ 25 - 50%
- ◯ 5 - 24%
- ◯ < 5%

At any given time, what percentage of your YLWH patients are pregnant?

- ◯ >75%
- ◯ 51 - 75%
- ◯ 25 - 50%
- ◯ 5 - 24%
- ◯ < 5%

What percentage of your YLWH patients have transportation challenges (e.g. no car, they do not drive, public transportation is difficult to access or pay for, they need support from your clinic to come to appointments) ?

- ◯ >75%
- ◯ 51 - 75%
- ◯ 25 - 50%
- ◯ 5 - 24%
- ◯ < 5%

What percentage of your YLWH patients have HOUSING challenges (e.g. unstably housed, living in a shelter, motel, with friends or family temporarily etc.) ?

- ◯ >75%
- ◯ 51 - 75%
- ◯ 25 - 50%
- ◯ 5 - 24%
- ◯ < 5%

For what percentage of your YLWH patients are you the primary care provider (i.e. the provider who manages non-HIV comorbidities in addition to their HIV)?

- ◯ >75%
- ◯ 51 - 75%
- ◯ 25 - 50%
- ◯ 5 - 24%
- ◯ < 5%

What percentage of your YLWH patients have limited English proficiency?

- ◯ >75%
- ◯ 51 - 75%
- ◯ 25 - 50%
- ◯ 5 - 24%
- ◯ < 5%

When communicating your YLWH patients who have limited English proficiency, what percentage of the time do you use a professional translator service or trained bilingual staff member?

- ◯ >75%
- ◯ 51 - 75%
- ◯ 25 - 50%
- ◯ 5 - 24%
- ◯ < 5%

What percentage of your YLWH patients do not have access to the EQUIPMENT necessary to conduct video visits? (e.g. smart phone, computer, or tablet etc.)

- ◯ >75%
- ◯ 51 - 75%
- ◯ 25 - 50%
- ◯ 5 - 24%
- ◯ < 5%

What percentage of your YLWH patients do not have access to the NETWORK necessary to conduct video visits? (e.g. access to wifi or adequate data plan.)	◯ >75%<br>◯ 51 - 75%<br>◯ 25 - 50%<br>◯ 5 - 24%<br>◯ < 5%

Current and Future Telehealth Use

Section 2/5

This section will assess your current telehealth usage along with the role you see for telehealth in the future for working with youth patients living with HIV (age 16-29).

Definitions of telehealth visits

Video visits - a billable visit that replaces an in-person visit that utilizes audio AND video technology. You can see your patient and they can see you. You are not in the same location as your patient.

Telephone visits - a billable visit that replaces an in-person or video visit that utilizes ONLY audio. This is different from a patient/provider follow-up phone call that is not billed as a visit. You are not in the same location as your patient.

In-person visits - a face to face visit that takes place in clinic or other health care setting. You are in the same location as your patient.

BEFORE the COVID-19 pandemic caused shelter in place orders in the U.S. (operationalized as March 15, 2020), what percentage of your visits with YLWH were conducted as VIDEO visits?	◯ >75%<br>◯ 51 - 75%<br>◯ 26 - 50%<br>◯ 5 - 25%<br>◯ < 5%
In 2020 and/or 2021, at the height of the COVID-19 pandemic in your area, what percentage of your visits with YLWH were conducted as VIDEO visits?	◯ >75%<br>◯ 51 - 75%<br>◯ 26 - 50%<br>◯ 5 - 25%<br>◯ < 5%
In the PAST 6 MONTHS, what percentage of your visits with YLWH were conducted as VIDEO visits?	◯ >75%<br>◯ 51 - 75%<br>◯ 26 - 50%<br>◯ 5 - 25%<br>◯ < 5%
BEFORE the COVID-19 pandemic caused shelter in place orders in the U.S. (operationalized as March 15, 2020), what percentage of your visits with YLWH were conducted as TELEPHONE visits?	◯ >75%<br>◯ 51 - 75%<br>◯ 26 - 50%<br>◯ 5 - 25%<br>◯ < 5%
In 2020 and/or 2021, at the height of the COVID-19 pandemic in your area, what percentage of your visits with YLWH were conducted as TELEPHONE visits?	◯ >75%<br>◯ 51 - 75%<br>◯ 26 - 50%<br>◯ 5 - 25%<br>◯ < 5%

In the past 6 MONTHS, what percentage of your visits with YLWH were conducted as TELEPHONE visits?	◯ >75%<br>◯ 51 - 75%<br>◯ 26 - 50%<br>◯ 5 - 25%<br>◯ < 5%
Which of the following factors contribute to which care modality (phone, video, or in-person) you use with your YLWH patients? Choose all that apply.	☐ My clinical judgment<br>☐ Patient preference<br>☐ Scheduler preference/messaging<br>☐ Leadership/institutional guidance<br>☐ Public health guidance<br>☐ Workload credit<br>☐ Available data/research comparing effectiveness of the modalities<br>☐ Technical or connectivity concerns<br>☐ Other
If "other", please specify:	__________________________
In your IDEAL practice, what percentage of visits do you conduct virtually using telehealth?	0% ——————— 100%<br>*(Place a mark on the scale above)*
For measuring retention in HIV care for YLWH, do you think that a VIDEO visit should count equally as an in-person visit?	◯ Yes, under any circumstance<br>◯ Yes, but only for certain patients (e.g. patients with an undetectable VL)<br>◯ No, under no circumstance
Please describe which patients you believe a video visit should count as retention in care.	__________________________
If you offer telehealth visits for new patient visits, how soon after the initial visit do you see them in person?	◯ Within 1 week<br>◯ Within 1 month<br>◯ Within 3 months<br>◯ I do not see patients for initial visits via Telehealth
Who do you offer telehealth visits to?	◯ Patients who are MORE engaged-in-care<br>◯ Patients who are LESS engaged-in-care<br>◯ Everyone equally<br>◯ No one, we do not offer Telehealth visits
How do you include telehealth via video in your clinical HIV practice for YLWH? Check all that apply.	☐ We have a protocol where we see patients via telehealth intermittently as long as we can still have an in person visit at least once or twice a year, which includes obtaining labs, STI testing, and other clinical procedures as per standard of care.<br>☐ We do not have a specific protocol but we see patients as needed via telehealth as long as we can obtain labs, STI testing or other clinical procedures as per standard of care<br>☐ Generally, no one is offered telehealth and it is only used in rare circumstances<br>☐ We do not have an option for telehealth<br>☐ Other (please describe)

If other, please describe

____

Considering your youth patients (YLWH), how much do you agree or disagree with the following statements regarding the future of VIDEO visits?

	Strongly disagree	Disagree	Neutral	Agree	Strongly agree
Telehealth will play a role in the future of HIV care	○	○	○	○	○
Patients who use video telehealth are more engaged in care through both medication adherence and being seen by a provider more often	○	○	○	○	○
Telehealth has the potential to improve my daily workflow/efficiency.	○	○	○	○	○
Video visits for people living with HIV should be expanded	○	○	○	○	○
Assuming reimbursement stays the same video visits are here to stay	○	○	○	○	○
Moving forward we should scale back video visits	○	○	○	○	○

When conducting IN-PERSON visits with YLWH patients, how often do you discuss the following?

	Never	Rarely	Sometimes	Often	Always
Adherence to medications	○	○	○	○	○
Test results (e.g. labs, imaging)	○	○	○	○	○
Home and work life (e.g. housing status, life stressors)	○	○	○	○	○
Substance use (e.g. tobacco, alcohol, illicit drugs)	○	○	○	○	○
Mental health	○	○	○	○	○
Sexual health (e.g. number and gender of partners, use of condoms, counseling on sexually transmitted infections)	○	○	○	○	○

When conducting VIDEO visits with YLWH patients, how often do you discuss the following?

	Never	Rarely	Sometimes	Often	Always
Adherence to medications	○	○	○	○	○
Test results (e.g. labs, imaging)	○	○	○	○	○
Home and work life (e.g. housing status, life stressors)	○	○	○	○	○
Substance use (e.g. tobacco, alcohol, illicit drugs)	○	○	○	○	○
Mental health	○	○	○	○	○
Sexual health (e.g. number and gender of partners, use of condoms, counseling on sexually transmitted infections)	○	○	○	○	○

When conducting TELEPHONE visits with YLWH patients, how often do you discuss the following?

	Never	Rarely	Sometimes	Often	Always
Adherence to medications	○	○	○	○	○
Test results (e.g. labs, imaging)	○	○	○	○	○
Home and work life (e.g. housing status, life stressors)	○	○	○	○	○
Substance use (e.g. tobacco, alcohol, illicit drugs)	○	○	○	○	○
Mental health	○	○	○	○	○
Sexual health (e.g. number and gender of partners, use of condoms, counseling on sexually transmitted infections)	○	○	○	○	○

Training and Technical Issues

Section 3/5
This section will gather information about training and technical issues related to telehealth.

What type of telehealth training did you receive? Check all that apply.

- ☐ In person or digital classroom training
- ☐ Digital instructions via email, Powerpoint or PDF
- ☐ Pre-recorded videos provided by my institution
- ☐ Pre-recorded videos I found online (Youtube or other website)
- ☐ 1:1 help from a professional in my institution
- ☐ 1:1 help from a tech savvy friend or co-worker
- ☐ Other (please describe)
- ☐ I did not have any Telehealth training
- ☐ I did not have enough telehealth training
- ☐ I could benefit from additional training

If "other", please describe

____

How helpful was the training and/or information you received about the system installation and setup prior to conducting your first virtual video visit?

- ◯ Very helpful
- ◯ Somewhat helpful
- ◯ Not very helpful
- ◯ Not at all helpful
- ◯ I did not receive information about the system or help setting it up

Which modalities of telehealth are you currently using (choose all that apply)?

- ☐ VA Video Connect (VVC)
- ☐ Epic video visits
- ☐ Zoom (video + audio)
- ☐ Zoom (audio-only)
- ☐ Doximity (video + audio)
- ☐ Doximity (audio only)
- ☐ Doxy.me (video + audio)
- ☐ Doxy.me (audio only)
- ☐ VIDYO (video + audio)
- ☐ Other video visit software
- ☐ Telephone or Telephonic encounters

If "Other," please specify:

____

How many VIDEO visits did you conduct before you felt comfortable with the technology?

- ◯ 1-3 visits
- ◯ 4-10 visits
- ◯ More than 10 visits
- ◯ My comfort level varies with the device/technology the patient is using
- ◯ I am still not comfortable with the technology

What issues have you encountered during any VIDEO visits that you have conducted with YLWH patients? (check all that apply)

- ☐ Logging in
- ☐ Joining the visit (You)
- ☐ Joining the visit (Patient)
- ☐ Remaining connected during the visit
- ☐ Other providers and team members joining the visit
- ☐ Sound (hearing or being heard)
- ☐ Video (seeing or being seen)
- ☐ Ending the visit
- ☐ None of the above
- ☐ Other (please specify)

If other, please specify:

____

How often do you experience technical problems with virtual video visits?

- ◯ Always
- ◯ Often
- ◯ Sometimes
- ◯ Rarely
- ◯ Never

Are you able to troubleshoot on your own without technical assistance when technological issues arise on a telemedicine platform?

- ◯ Always
- ◯ Often
- ◯ Sometimes
- ◯ Rarely
- ◯ Never

Please provide any comments about technical issues you have encountered.

____

Have you received specific training on building rapport and maintaining patient-provider trust while using telehealth?

- ◯ Yes
- ◯ No

Do you think specific training designed to improve the personal patient-provider connection and trusted relationship would be beneficial to your future telehealth use?

- ◯ Yes
- ◯ No

If you could improve anything about the virtual visit experience, what would it be?

____

You are more than half way done!

Benefits and Barriers

Section 4/5 You are almost done! The following questions will assess the benefits and barriers of using telehealth with your YLWH patients.

The next 5 questions ask you to RANK in order your opinions on barriers and benefits of telehealth. In each section, every option must have a UNIQUE RANK. For example, if there are 6 options they must each be ranked 1 through 6 with 1 being the most beneficial and 6 being the least beneficial. No option can have the same rank as another. It may be helpful to read all the options before ranking.

Please RANK in order 1-6 the top PATIENT BENEFITS of telehealth with 1 being the most beneficial and 6 being the least beneficial.

	Most Beneficial 1 (First)	2	3	4	5	Least Beneficial6 (Last)
Decreases barriers (i.e. transportation, childcare, etc)	○	○	○	○	○	○
Patients prefer flexibility in having the choice of telehealth	○	○	○	○	○	○
Protects privacy being seen remotely vs. at an HIV clinic (may minimize HIV stigma)	○	○	○	○	○	○
Increased ability to build patient-provider trust	○	○	○	○	○	○
Protects the health of patients/staff	○	○	○	○	○	○
Saves time for the patient	○	○	○	○	○	○

Please RANK in order 1-6 the top PROVIDER BENEFITS of telehealth with 1 being the most beneficial and 6 being the least beneficial.

	Most Beneficial 1 (First)	2	3	4	5	Least Beneficial 6(Last)
Convenient for providers (e.g. remote work)	○	○	○	○	○	○
Improves clinic efficiency (e.g. saves time)	○	○	○	○	○	○
Fewer no-shows/canceled appts	○	○	○	○	○	○
Favorable compensation	○	○	○	○	○	○

Increases patient-provider trust	○	○	○	○	○	○
Protects the health of patients/staff	○	○	○	○	○	○

Please RANK in order 1-6 the top PATIENT BARRIERS of telehealth with 1 being the biggest barrier and 6 being the smallest barrier.

	Biggest Barrier 1 (First)	2	3	4	5	Smallest Barrier 6 (Last)
Technical difficulties on the patient end	○	○	○	○	○	○
Concerns about patient privacy (e.g. visits at home or work)	○	○	○	○	○	○
Unfavorable insurance coverage	○	○	○	○	○	○
Decreased ability to build trust with provider	○	○	○	○	○	○
Inability for patients to complete paperwork and meet with support staff (e.g. ADAP and insurance forms and meet with case manager)	○	○	○	○	○	○
Access to technology (e.g. smart phone, data plan, or wifi)	○	○	○	○	○	○

Please RANK in order 1-6 the PROVIDER BARRIERS of telehealth with 1 being the biggest barrier and 6 being the smallest barrier.

	Biggest Barrier 1 (First)	2	3	4	5	Smallest Barrier 6 (Last)
Challenges building patient-provider trust	○	○	○	○	○	○
Inability to perform a complete physical exam	○	○	○	○	○	○
Challenges obtaining labs, STI tests, or other clinical tests	○	○	○	○	○	○
Providing vaccines	○	○	○	○	○	○
Obtaining vital signs in clinic	○	○	○	○	○	○
Patient may be distracted or not in an appropriate setting	○	○	○	○	○	○

Please RANK in order 1-5 the SYSTEM LEVEL BARRIERS of telehealth with 1 being the biggest barrier and 5 being the smallest barrier.

	Biggest Barrier 1 (First)	2	3	4	Smallest Barrier 5 (Last)
Technical difficulties	○	○	○	○	○
Switching between in-person and virtual visits impacts clinic efficiency	○	○	○	○	○
Increased no-shows/canceled appointments	○	○	○	○	○
Unfavorable compensation or insurance coverage	○	○	○	○	○
Not enough support/training from leadership or organization	○	○	○	○	○

For each of the following, indicate if a VIRTUAL (video or phone) visit is better, an in-person/office visit is better, or there is no difference

	Virtual is better	No difference	In-person is better	Does not apply
Workflow outside of the visit (before and after)	○	○	○	○
Appropriate length of visit to assess and treat patient	○	○	○	○
Ease of scheduling followup visits	○	○	○	○
Quality of patient--provider communication	○	○	○	○
The ability to see and assess a physical problem	○	○	○	○
Patient adherence to treatment plans	○	○	○	○
Personal connection I feel with the patient	○	○	○	○
Ease of accessing records and ordering tests during the visit	○	○	○	○
Efficiency of the visit	○	○	○	○
Overall patient quality is improved	○	○	○	○
Building patient-provider trust	○	○	○	○
Discussing sensitive topics and delivering serious news	○	○	○	○

Compared to IN-PERSON visits...

	Strongly disagree	Disagree	Neutral	Agree	Strongly agree
Video visits increase patient privacy	○	○	○	○	○
Video visits improve quality of patient care	○	○	○	○	○
Video visits reduce patients' anxiety about being seen at an HIV clinic	○	○	○	○	○
Video visits increase the number of times I can interact with my patients	○	○	○	○	○
Video visits improve access to care	○	○	○	○	○
Video visits reduce the amount of time patient spend getting to/from visits	○	○	○	○	○
Video visits allow patients to feel more comfortable being open with their provider	○	○	○	○	○
Video visits take less time for the patients than in person visits	○	○	○	○	○

Did you shift to using telehealth for the following clinic activities? (Please check all that apply)

- ☐ Research
- ☐ Community Advisory Boards
- ☐ Support Groups
- ☐ Patient Educational Activities
- ☐ Other
- ☐ None of the Above

If "Other" please describe:

____

Are you still using telehealth for any of the following clinic activities? (Check all that apply)

- ☐ Research
- ☐ Community Advisory Boards
- ☐ Support Groups
- ☐ Patient Educational Activities
- ☐ Other
- ☐ None of the Above

If "Other" please describe:

____

Thanks for completing this section. The final section only has four short questions and then you are done!

Open Ended Questions

Section 5/5

Please provide us with a little more information regarding your thoughts and opinions on Telehealth use, including best practices and silver linings.

133) Please share any tools, tips, or helpful ways you have navigated the shift to telehealth, including creative ways to manage the challenges faced not being able to do an in person physical exam, labs, STI testing or other medical procedures and tests. __________________________________

134) Please describe strategies you have used to build and maintain patient-provider trust while using Telehealth. __________________________________

135) Please describe a silver lining for YOU related to electronic health care. (e.g. telehealth, patient access to electronic medical records (EMR), mobile health apps, and/or the ability for patients to message providers, make appointments etc. electronically.) __________________________________

If there is no silver lining - please ket us know.

136) Please describe a silver lining for YOUR PATIENTS (YLWH) related to electronic health care. (e.g. telehealth, patient access to electronic medical records (EMR), mobile health apps, and/or the ability for patients to message providers, make appointments etc. electronically.) __________________________________

If there is no silver lining - please ket us know.

Thank you for completing the survey!

Next you will provide your email for your gift card.

Thank you for your participation

Thank you again for taking the time to provide valuable information to help us learn more about ways to engage YLWH in care. You will be compensated $25 for your time. This will be sent to you via email within the next 2 weeks.

Your email will be used to send you the electronic gift card and will not be connected to the data provided on the survey.

If you refer two or more people who complete the survey, and they provide your email at the end, you will recieve gift card(s) for helping us recruit!

---

137) Please provide your work email which will be verified and used for compensation.

*If you are supplying a gmail or other non-work email we will need to verify your place of employment before compensating you, as you must meet eligibility criteria to participate in the survey.

____

---

138) What is the name of your clinic? (This data will not be published or shared)

____

---

139) Would you be willing to be contacted about future research related to HIV prevention and care?

◯ Yes
◯ No

---

140) Would you like to be informed of any publications related to this study?

◯ Yes
◯ No

---

141) Please provide the email address of the person that referred you to this study. They will be compensated with a gift card for referrals.

*No need to provide the email if it was a study team member, i.e. Emily Barr, Robin Hardwicke, Mary Paul, Hannah Bernath, or Tom Giordano

**If you refer two people or more who complete the survey, you will also be compensated with a gift card. Please share the link.

____
